# Maternal and Infant Supplementation with Small-Quantity Lipid-Based Nutrient Supplements Increases Infants’ Iron Status at 18 Months of Age in a Semiurban Setting in Ghana: A Secondary Outcome Analysis of the iLiNS-DYAD Randomized Controlled Trial

**DOI:** 10.1093/jn/nxy225

**Published:** 2019-01-08

**Authors:** Seth Adu-Afarwuah, Rebecca T Young, Anna Lartey, Harriet Okronipa, Per Ashorn, Ulla Ashorn, Brietta M Oaks, Mary Arimond, Kathryn G Dewey

**Affiliations:** 1 Department of Nutrition and Food Science, University of Ghana, Legon, Accra, Ghana; 2 Program in International and Community Nutrition, Department of Nutrition, University of California, Davis, CA; 3 Center for Child Health Research, University of Tampere and Tampere University Hospital, Tampere, Finland; 4 Department of Nutrition and Food Sciences, University of Rhode Island, Kingston, RI; 5 *Intake—*Center for Dietary Assessment, FHI 360, Washington, DC

**Keywords:** micronutrient supplementation, iron and folic acid, multiple micronutrient supplements, small-quantity lipid-based nutrient supplements, infants

## Abstract

**Background:**

Interventions are needed to address iron deficiency in low-income settings.

**Objective:**

This secondary outcome analysis aimed to compare the hemoglobin (Hb) and iron status [zinc protoporphyrin (ZPP)] of children born to women enrolled in the iLiNS-DYAD trial in Ghana.

**Methods:**

Women ≤20 wk pregnant (*n* = 1320) were assigned to receive 60 mg Fe/d and 400 µg folic acid/d until delivery and placebo thereafter, and no supplementation for infants (IFA group); or multiple micronutrients containing 20 mg Fe/d until 6 mo postpartum and no supplementation for infants (MMN); or small-quantity lipid-based nutrient supplements (SQ-LNSs) containing 20 mg Fe/d until 6 mo postpartum, and SQ-LNSs for infants from 6 to 18 mo of age (LNS). We compared infants’ Hb (g/L) and ZPP (µmol/mol heme) at 6 and 18 mo of age.

**Results:**

At 6 mo of age, groups did not differ in mean ± SD Hb (overall: 113 ± 9.9 g/L) or geometric mean (95% CI) ZPP [overall: 62.6 (60.6, 64.7)]. At 18 mo of age, mean ± SD Hb (overall: 112 ± 10.4 g/L) did not differ significantly between groups, whereas geometric mean (95% CI) ZPP was lower (*P* = 0.031) in the LNS group [53.9 (50.7, 57.3)] than the IFA [60.4 (56.7, 64.3)] but not the MMN [58.8 (55.6, 62.2)] group. Further, the LNS group, compared with the IFA and MMN groups combined, had a lower prevalence of elevated (>70) ZPP (27.5% compared with 35%; *P* = 0.02) and a marginally lower prevalence of anemia (38.7% compared with 44.9%; *P* = 0.06). These results generally remained unchanged when controlling for prespecified covariates or correcting for inflammation.

**Conclusions:**

In this setting, providing SQ-LNSs or multiple micronutrients with 20 mg Fe/d, compared with iron (60 mg/d) and folic acid, to pregnant women does not affect their infants’ Hb or iron status at 6 mo of age, but maternal and infant supplementation with SQ-LNSs increases infants’ iron status at 18 mo of age.

This trial was registered at clinicaltrials.gov as NCT00970866.

## Introduction

Inadequate micronutrient intake is common in low-income countries (1) and has been associated with increased risk of perinatal morbidity and mortality (2), low birth weight (3), and poor child growth and development (4). Consequently, addressing the micronutrient needs of women and children especially during the “first 1000 d” is a global priority (5–7).

As part of the International Lipid-based Nutrient Supplements (iLiNS) Project, we developed small-quantity lipid-based nutrient supplements (SQ-LNSs), which can be used to enrich home-prepared foods for women and children (8), and thereby increase the intakes of essential nutrients without causing substantial changes in usual dietary practices. During the last several years, similar or different formulations of SQ-LNS products (9) have been used in many intervention trials and programs worldwide (10).

In the iLiNS-DYAD randomized, controlled supplementation trial in Ghana, we evaluated the efficacy of SQ-LNSs given to women during pregnancy and the first 6 mo postpartum, and to their offspring from 6 to 18 mo of age, based on evidence that child malnutrition in developing countries often begins in utero and continues after birth (11). We previously reported the primary growth outcomes (12, 13) as well as several other secondary outcomes (14–18) of the trial and showed that maternal–infant supplementation with SQ-LNSs, compared with maternal-only supplementation with iron and folic acid or multiple micronutrients, promoted infant and child growth in our trial setting.

In West Africa, an estimated 80% of children <5 y of age are anemic (19), up to one-half of which are estimated to be due to iron deficiency (20). Possible consequences of iron deficiency anemia (IDA) include cognitive impairment in children (21) and lower school performance (22). In this present analysis of secondary outcomes of the trial, we aimed to determine the impact of the iLiNS-DYAD intervention on children's blood hemoglobin (Hb) and iron status and inflammation biomarkers at 6 and 18 mo of age.

## Methods

### 

#### Study setting, design, and participants

The iLiNS-DYAD Ghana trial (NCT00970866) has been described in detail previously (12–14). Briefly, the trial was conducted in the Somanya–Odumasi–Kpong area, a semiurban setting ∼70 km north of Accra, and was designed as a partially double-blind, individually randomized, controlled trial with 3 equal-size groups. Women ≥18 y old and ≤20 wk pregnant identified from antenatal clinics were recruited after obtaining informed consent for themselves and for their infants upon delivery. Exclusion criteria were: not considered as a resident of the area, intention to move out of the area, milk or peanut allergy, participation in another trial, HIV infection, asthma, epilepsy, tuberculosis, any malignancy, or unwillingness to receive fieldworkers or take the study supplement.

The trial was approved by 3 ethics committees (University of California, Davis; Ghana Health Service; and Noguchi Memorial Institute for Medical Research) and monitored by a Data and Safety Monitoring Board.

#### Group assignments and blinding

As reported previously (12–14), recruited women who remained eligible were, after baseline assessments, randomly assigned to consume 60 mg Fe + 400 µg folic acid/d (IFA supplement or group), multiple micronutrients containing 18 vitamins and minerals (MMNs supplement or group), or 20 g SQ-LNS/d (LNS group) during pregnancy. In the first 6 mo postpartum, women in the IFA group were assigned 200 mg Ca/d as placebo, and those in the MMN and LNS groups assigned the same supplements as during pregnancy. From 6 to 18 mo of age, infants born to women in the IFA and MMN groups were assigned to receive no micronutrient supplementation, whereas those of women in the LNS group were assigned to consume SQ-LNSs designed for infants. The IFA and MMN supplements were provided as capsules in blister packs of 10, whereas the SQ-LNS for women was in 20-g sachets, and that for infants in 10-g sachets (given 2/d).

The following individuals completed the group assignments (12, 13, 17): *1*) the Study Statistician at UC Davis, Janet M Peerson, developed the allocations in blocks of 9 (SAS for Windows version 9.4); *2*) someone at the University of Ghana not involved in the recruitment of subjects prepared the envelopes containing the assignments, which were numbered and stacked by block number; and *3*) the Study Nurse at the field site performed the random assignment. At each enrollment, the Study Nurse shuffled 9 envelopes taken from the top of the stack and asked the participant to make a pick to reveal the assignment. The Nurse then returned the unused envelopes to the top of the stack. When there were <9 women left to be enrolled, the Nurse shuffled whatever number of envelopes remained. Any allocation information was kept securely by the Field Supervisor in Ghana and the Study Statistician at UC Davis only.

At enrollment, the Study Nurse gave women a 2-wk supply of supplements, advice to take 1 capsule/d with water after a meal, or one 20-g SQ-LNS sachet/d mixed with food, and a standard nutrition message about the need to “eat meat, fish, eggs, fruits, and vegetables” whenever possible (12, 13). The IFA and MMN capsules were known to the study team and participants only by their color codes (3 colors for IFA and 3 for MMN). It was not possible to blind study workers and participants to the capsules and the LNSs owing to their apparent differences, but laboratory staff and data analysts had no knowledge of the group assignments.

#### Follow-up

During pregnancy, fieldworkers visited women in their homes biweekly to deliver supplements. All live-born singleton infants delivered by the women were enrolled into the study. In cases of multiple births, only 1 infant was selected randomly, and the selected infant was enrolled if he or she was born alive. After women gave birth, fieldworkers visited them and their infants weekly, but delivered the women's supplements or placebo biweekly as before.

Women exited the study at 6 mo postpartum, but the weekly visits for the infants continued. At 6 mo of age, the Study Nurse delivered the “minimum message” on complementary feeding to all mothers at the laboratory after the infants’ first laboratory assessment: “Breastfeed your baby as you did before 6 mo of age; do not forget to feed your baby meat, fish, eggs, fruits and vegetables whenever you can.” During the next usual weekly home visit, fieldworkers delivered the first supply of SQ-LNSs for infants in the LNS group, advised those mothers or caregivers on how to feed the supplements (i.e., mix the entire content of 1 sachet with 2–3 tablespoons of food for the infant before feeding additional foods if the infant desires, 2 times/d), and repeated the “minimum message” on complementary feeding given by the Study Nurse. For infants in the IFA and MMN groups who were not assigned to any supplements, fieldworkers also repeated the “minimum message” to their mothers or caregivers. Fieldworkers delivered a fresh supply of infants’ SQ-LNSs during the usual weekly visits, but the “minimum message” was not repeated again thereafter. Infants exited the study at 18 mo of age.

#### Trial supplements

As previously reported (8, 12–14, 16), the IFA reflected the Ghana Health Service's standard micronutrient supplementation for pregnant women in Ghana at the time of the study (23). The daily dose of MMN contained 18 vitamins and minerals at 1 or 2 times the RDA for pregnancy, except iron. For iron, we used 20 mg/d instead of the 30 mg/d in the UNICEF/WHO/United Nations University International Multiple Micronutrient Preparation (UNIMMAP) formulation (24) or the 30–60 mg/d in the WHO guideline (23), because of previous evidence (25) showing that 20 mg Fe/d was just as effective as 40 mg/d or 80 mg/d in treating anemia during pregnancy and was likely to cause fewer gastrointestinal side effects compared with a 30–60 mg/d dosage. The SQ-LNS for women had similar micronutrient contents to the MMN, plus energy, protein, and essential fatty acids as well as the maximum amounts of calcium, magnesium, phosphorus, and potassium that could be included given technical and organoleptic constraints. Including 20 mg Fe/d in the SQ-LNS for women made it possible to have just one product for both pregnancy and lactation, assuming that this amount, in addition to iron from the usual diet, would give a total daily intake that was close to the amount in the UNIMMAP formulation (24) and would therefore meet the RDA of 27 mg Fe for pregnancy, while at the same time not greatly exceeding the RDA (9 mg/d) for lactation (8).

The SQ-LNS for infants was designed to generally supply the WHO/FAO Recommended Nutrient Intake for key micronutrients for infants 7–12 mo of age, with a few exceptions (8). We used an iron dosage (6 mg/d) that was approximately the WHO/FAO Recommended Nutrient Intake for infants 7–12 mo of age when assuming high bioavailability (6.2 mg/d), and 33% lower than the dosage previously used in Ghana (26), because of concerns about increasing the risk of malaria and infections in an area of high malaria endemicity (27). Zinc content (8 mg/d) was kept higher (8, 28) than the 4 mg/d used in our previous study (26) because zinc absorption may be decreased in the study area's predominantly maize-based diet high in phytate.

#### Data collection

Women's baseline assessments included sociodemographic characteristics; gestational age (by using ultrasound biometry, Aloka SSD 500); anthropometric status [by using standard procedures (29)]; venous blood Hb concentration (HemoCue Hb301; Hemocue AG); zinc protoporphyrin concentration (ZPP) (Hematofluorometer; Aviv Biomedical Co.); and peripheral malaria parasitemia (Vision Biotech) (13). Hb concentration was measured within 2 min after the blood draw. We used the original Aviv cover-slides and 3-level control material for the ZPP measurements, after red blood cells were washed 3 times with normal saline. Women's supplement intakes were monitored biweekly by collecting unused blister packs or SQ-LNS sachets at each visit. In addition, during the biweekly interviews, women were asked on how many days since the last visit they had consumed the supplements, reasons (if any) they did not consume the supplements, and, for those in the LNS group, whether someone else had consumed some of the supplements. Any discrepancies between the responses to these questions and the number of capsules or sachets remaining since the last visit were resolved during the interview.

For infants in the LNS group, supplement intakes were monitored weekly by training mothers to use a calendar grid to indicate, on a daily basis, whether infants consumed the supplement. Calendar grids were checked at each weekly visit. Mothers unable to use the calendar grid were asked to recall the children's supplement intakes for each day since the last visit. In addition, fieldworkers collected any unused SQ-LNS sachets at each visit, and reconciled the number of sachets remaining since the last visit with the consumption information recorded on the calendar grid or provided by the mother. At 6 and 18 mo of age, infants were brought to the laboratory, where we collected venous blood, and measured Hb concentration within 2 min, as well as measuring ZPP concentrations and peripheral malaria parasitemia for all infants using the same techniques used for the women. The infants’ plasma samples obtained after centrifuging the blood at 1252 × *g* for 15 min at a room temperature of ∼23°C were stored in Ghana at −33°C, before being air-freighted on dry ice to the USDA Western Human Nutrition Research Center (WHNRC) in Davis, CA, where we determined the C-reactive protein (CRP) and α-1 glycoprotein (AGP) concentrations in a randomly selected subsample of infants by using a Cobas Integra 400 plus Automatic Analyzer (Roche Diagnostic Corp.).

The secondary outcomes evaluated herein were infants’ Hb (grams per liter), ZPP (micromoles per mole of heme), CRP (milligrams per liter), and AGP (grams per liter) concentrations at 6 and 18 mo of age, and the percentages of infants with anemia (indicated by low Hb), iron deficiency (indicated by elevated ZPP), and IDA (low Hb plus elevated ZPP) at 6 and 18 mo of age.

#### Sample size and data analysis

As described previously (12, 13), the sample size for the iLiNS-DYAD-Ghana study was based on detecting an effect size or Cohen's *d* (30) of 0.3 between any 2 groups for any continuous variables, with a 2-sided 5% test and 80% power. We previously reported the temporary mislabeling of some IFA and MMN supplements (12, 13), as a result of which 170 women in the IFA group inadvertently received MMN capsules either throughout (*n* = 85) or during part of (*n* = 85) their pregnancy before receiving the intended IFA capsules, and 170 women in the MMN group received IFA capsules either throughout (*n* = 78) or during part of (*n* = 92) their pregnancy before receiving the intended MMN capsules. In total, 1320 women were enrolled.

This present analysis includes all of the children for whom data were available, consistent with our analysis of the primary growth outcomes (12). For CRP and AGP analyses (as well as several other biochemical outcomes in the trial), however, the target sample size was based on detecting an effect size of ≥0.5 between any 2 groups, with a 2-sided 5% test and 80% power. This required 105 subjects/group, or 315 subjects for the 3 groups, after taking into account ≤25% attrition. The subsample for the CRP and AGP analyses was selected from the children whose mothers had not been pregnant during the period when the temporary mislabeling occurred.

The analysis reported herein was part of the iLiNS-DYAD-Ghana statistical analysis plan, which we posted at our website (www.ilins.org) before data analysis began. Both the statistical analysis plan and the trial protocol at clinicaltrials.org listed children's blood Hb and iron status as secondary outcomes to be analyzed separately from the primary outcomes. We used Hb <100 g/L to define anemia in women (14, 31, 32). For children, we defined anemia by using the cutoff of Hb <110 g/L according to the WHO (32, 33), as well as by the cutoff values of Hb <105 g/L for 6 mo of age described by Domellöf et al. (34), and Hb <100 g/L for 18 mo of age (31) described by the International Nutritional Anemia Consultative Group (INACG), because of concerns that the WHO cutoff may be set too high. We defined elevated ZPP as ZPP >70 µmol/mol heme (35–37); elevated CRP as CRP >5.0 mg/L (38); and elevated AGP as AGP >1.0 g/L (38). Infants’ Hb values were considered to be normally distributed, but the ZPP, CRP, and AGP values (Shapiro–Wilk W <0.95 in all cases) were ln-transformed before analysis.

We analyzed data on an intention-to-treat basis (by including children regardless of adherence to treatment), using SAS for Windows version 9.4 (SAS Institute). Background maternal and household variables were summarized by intervention groups based on supplements women were *intended* to receive when enrolled. Because of the protocol violation associated with the consumption of mislabeled IFA or MMN capsules, we analyzed our data by using 2 scenarios, as done previously (12): in the first, intervention groups were based on the supplements women were intended to receive when enrolled; in the second, groups were based on the supplements women actually received when enrolled. In addition, we performed a secondary analysis by using a 2-group comparison in which the IFA and MMN groups were combined and compared with the LNS group (12).

We analyzed continuous and binary outcomes at 6 and 18 mo of age, by using linear and logistic regression models (SAS, PROC GLIMMIX), with Tukey–Kramer adjustment for multiple comparisons. We compared group means ± SDs (or SEs) or geometric means (95% CIs) for continuous outcome measures, and group percentages (95% CIs) for binary outcomes. Along with group comparisons, we estimated contrasts between groups (effect size) and their 95% CIs (39), including difference in means and ratio of log-transformed means for continuous outcomes, and RRs (40) for binary outcomes.

Both unadjusted and adjusted analyses were performed. In the adjusted analyses, only prespecified potential covariates (maternal age, height, BMI, education, gestational age at enrollment, primiparity, season at enrollment, baseline anemia status, and proxy indicators for household socioeconomic status and child sex) significantly associated with the outcome at α = 0.1 in bivariate analyses were included in the final models. For binary outcomes, the adjusted percentages were generated by using the technique described by Kleinman and Norton (41).

In the subsample of children for whom there were CRP and AGP data, we performed a sensitivity analysis as done previously (14), by correcting for the effect of inflammation (CRP and AGP) on the Hb and iron status outcomes (38) and repeating the above analyses. We used 3 inflammation categories, namely: reference (normal CRP and AGP), incubation (raised CRP and normal AGP), and early (raised CRP and AGP) or late (normal CRP and raised AGP) convalescence, to calculate the corrected Hb and ZPP values (44).

We have presented statistics in the text as means ± SDs or geometric means (95% CIs) for continuous outcomes, and percentages for binary outcomes. As we published previously, the self-reported adherence to supplement intake for women (pregnancy/lactation) was 88.1%/85.7% for the IFA group, 87.0%/85.0% for the MMN group, and 83.7%/80.0% for the LNS group (45), and that for the infants in the LNS group was 73.5% (12).

## Results

Data were collected between December 2009 and March 2014. In total, 1320 women were enrolled. At baseline, the women were ∼27 y of age on average, had ∼8 y of formal education, and were at ∼16 weeks of gestation (**Table 1**); 10% tested positive in the rapid diagnostic test for malaria, and 14% had Hb <100 g/L.

**TABLE 1:** Characteristics of women (*n* = 1320) enrolled in the iLiNS-DYAD-Ghana nutrient supplementation trial, by group according to the supplements women were intended to receive when enrolled^1^

Background characteristics	IFA (*n* = 441)	MMN (*n* = 439)	LNS (*n* = 440)
Age, y	27 ± 5 (441)	27 ± 6 (439)	27 ± 6 (440)
Formal education, y	8 ± 4 (441)	8 ± 4 (439)	8 ± 4 (440)
Gestational age at enrollment, wk	16.2 ± 3.3 (438)	16.0 ± 3.2 (438)	16.1 ± 3.3 (435)
Asset index^2^	0.05 ± 1.01 (433)	0.05 ± 0.99 (431)	–0.09 ± 1.00 (432)
Housing index^2^	0.05 ± 0.98 (433)	–0.03 ± 1.02 (431)	–0.01 ± 1.00 (432)
HFIAS score^3^	2.8 ± 4.6 (436)	2.4 ± 4.1 (429)	2.6 ± 4.0 (432)
Married or cohabiting, *n/N* (%)	406/441 (92.1)	413/439 (94.1)	405/440 (92.0)
Primiparous women, *n/N* (%)	162/441 (36.7)	137/439 (31.2)	147/440 (33.4)
Positive for malaria at baseline,^4^*n/N* (%)	40/441 (9.1)	39/438 (8.9)	54/440 (12.3)
Positive for malaria at 36 wk,^4^*n/N* (%)	30/348 (8.6)	34/362 (9.4)	38/336 (11.3)
Anemic at baseline,^5^*n/N* (%)	55/441 (12.5)	70/438 (16.0)	60/440 (13.6)
Anemic at 36 wk,^5^*n/N* (%)	13/349 (3.7)	23/362 (6.4)	27/338 (8.0)

1 Values are means ± SDs (*N*) or *n/N* (%). HFIAS, Household Food Insecurity Access Scale; IFA, Iron and Folic Acid group randomly assigned to receive 60 mg Fe/d and 400 mg folic acid/d during pregnancy and 200 mg Ca/d as placebo during the first 6 mo postpartum; iLiNS, International Lipid-based Nutrient Supplements; LNS, Lipid-based Nutrient Supplement group randomly assigned to receive 20 g small-quantity Lipid-based Nutrient Supplements/d with the same micronutrients as the MMN group, plus 4 more minerals (calcium, phosphorus, potassium, and magnesium) and macronutrients until 6 mo postpartum; MMN, Multiple Micronutrient group randomly assigned to receive 18 vitamins and minerals, including 20 mg Fe, daily until 6 mo postpartum; *n*, number of participants identified as “yes” for the variable in question; *N*, total number of participants in the group in question.

2 Proxy indicators for household socioeconomic status; higher values represent higher socioeconomic status.

3 HFIAS score is a proxy indicator for household food insecurity (42); higher values represent higher food insecurity.

4 Rapid diagnostic test (Clearview Malarial Combo; Vision Biotech).

5 Anemia defined as blood hemoglobin concentration <100 g/L (31, 32, 43).

**FIGURE 1:**
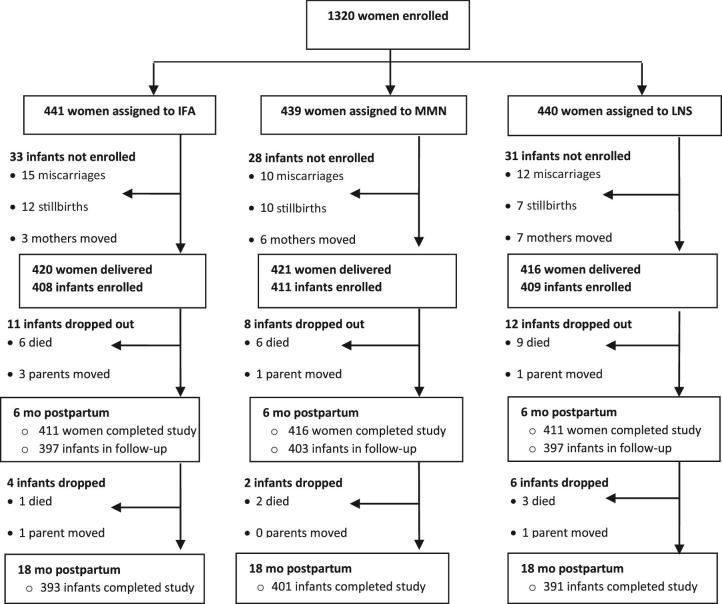
Study profile showing infants whose mothers were enrolled into the trial, and the reasons some infants were lost to follow-up. IFA, Iron and Folic Acid group: infants were assigned to receive no supplements, their mothers were assigned to receive 60 mg Fe/d and 400 µg folic acid/d during pregnancy and 200 mg Ca/d as placebo during the first 6 mo postpartum; LNS, Lipid-based Nutrient Supplement group: infants were assigned to receive 20 g Lipid-based Nutrient Supplement/d (designed for infants) containing 6 mg Fe/d from 6 to 18 mo of age, their mothers received 20 g Lipid-based Nutrient Supplement/d (designed for women) with the same micronutrients as the MMN group during pregnancy and the first 6 mo postpartum—both Lipid-based Nutrient Supplement products contained 4 additional minerals (calcium, phosphorus, potassium, and magnesium) as well as macronutrients; MMN, Multiple Micronutrient group: infants were assigned to receive no supplements, their mothers were assigned to receive a multiple micronutrient capsule containing 18 vitamins and minerals, including 20 mg Fe, daily during pregnancy and the first 6 mo postpartum.

We enrolled 1228 infants at birth out of 1257 deliveries; infants from 29 deliveries who were stillborn could not be enrolled (**Figure 1**). In all, 1197 infants attended the laboratory visit at 6 mo of age, and 1185 of the 1228 enrolled completed the study at 18 mo of age. The reasons some infants did not complete the study were death (*n* = 27), parental refusal (*n* = 9), and relocation from the study site (*n* = 7). Only 1.4% of infants at 6 mo of age and 1.1% at 18 mo of age had positive results in the rapid diagnostic test for malaria; these results did not differ by group at either of the 2 time points.

Overall, the children's mean ± SD Hb concentration was 113 ± 9.9 g/L at 6 mo of age and 112 ± 10.4 g/L at 18 mo of age, whereas the geometric mean (95% CI) ZPP concentration was 62.6 µmol/mol heme (60.6, 64.7 µmol/mol heme) at 6 mo of age and 57.6 µmol/mol heme (55.7, 59.7 µmol/mol heme) at 18 mo of age. When using the WHO cutoff (32, 33), the prevalence of anemia was 35.3% at 6 mo of age and 42.9% at 18 mo of age, whereas the prevalence of IDA was 19.1% at 6 mo of age and 21.0% at 18 mo of age. When using the cutoffs described by Domellöf et al. (34) and the INACG (31), however, the overall prevalences of anemia (18.6% at 6 mo of age; 5.2% at 18 mo of age) and IDA (11.9% at 6 mo of age; 3.9% at 18 mo of age) were much lower.

### 

#### Group comparisons

In the unadjusted analyses of continuous outcomes (**Table 2**), mean Hb and geometric mean ZPP concentrations at 6 mo of age did not differ by intervention group, but geometric mean CRP and AGP were significantly lower in the IFA and LNS groups than in the MMN group. At 18 mo of age, mean Hb and geometric mean CRP and AGP concentrations did not differ by intervention group, but geometric mean ZPP concentration was lower in the LNS group compared with the IFA, but not the MMN group, whereas the IFA and MMN groups did not differ in any of these outcomes. In the secondary analysis in which the IFA and MMN groups were combined (**Supplemental Table 1**), geometric mean (95% CI) ZPP concentration at 18 mo of age was significantly lower (*P* = 0.008) in the LNS group [53.9 µmol/mol heme (50.7, 57.3 µmol/mol heme)] than in the IFA + MMN group [59.6 µmol/mol heme (57.1, 62.1 µmol/mol heme)].

**TABLE 2:** Unadjusted continuous outcome measures (hemoglobin and biomarkers of iron status and inflammation) for infants in the iLiNS-DYAD randomized trial of daily nutrient supplementation in a semiurban setting in Ghana, by intervention group^1^

	Intervention group based on supplements mothers were intended to receive when enrolled				Intervention group based on supplements mothers actually received when enrolled			
Outcome variable	IFA (*n* = 441)	MMN (*n* = 439)	LNS (*n* = 440)	*P* ^2^	IFA (*n* = 441)	MMN (*n* = 439)	LNS (*n* = 440)	*P* ^2^
Hemoglobin, g/L								
6 mo	113 ± 11 (310)	113 ± 10 (325)	114 ± 10 (313)	0.72	114 ± 10 (308)	112 ± 10 (327)	114 ± 10 (313)	0.17
18 mo	112 ± 11 (333)	112 ± 11 (328)	113 ± 10 (328)	0.21	112 ± 11 (321)	112 ± 10 (340)	113 ± 10 (328)	0.19
ZPP, µmol/mol heme								
6 mo	62.8 (59.1, 66.7) [300]	61.5 (58.3, 65.0) [316]	63.5 (60.0, 67.1) [299]	0.49	60.8 (57.3, 64.5) [301]	63.4 (60.0, 67.1) [315]	63.5 (60.0, 67.1) [299]	0.74
18 mo	60.4 (56.7, 64.3)^b^ [329]	58.8 (55.6, 62.2)^ab^ [323]	53.9 (50.7, 57.3)^a^ [320]	0.031	59.6 (56.2, 63.3)^b^ [317]	59.5 (56.0, 63.1)^ab^ [335]	53.9 (50.7, 57.3)^a^ [320]	0.026
CRP, mg/L								
6 mo	0.32 (0.22, 0.46)^a^ [102]	0.65 (0.43, 0.99)^b^ [100]	0.41 (0.28, 0.60)^ab^ [101]	0.033	—	—	—	
18 mo	0.58 (0.39, 0.84) [101]	0.71 (0.50, 1.01) [101]	0.94 (0.63, 1.42) [100]	0.19	—	—	—	
AGP, g/L								
6 mo	0.80 (0.75, 0.86)^ab^ [102]	0.90 (0.84, 0.97)^b^ [100]	0.80 (0.74, 0.86)^a^ [101]	0.027	—	—	—	
18 mo	0.96 (0.89, 1.04) [101]	0.94 (0.88, 1.00) [101]	1.03 (0.96, 1.11) [100]	0.17	—	—	—	

1 All supplements were intended for daily consumption. The subsample for the CRP and AGP analyses was selected from the children whose mothers were not pregnant during the period when the temporary mislabeling occurred. Results are based on ANOVA (SAS PROC GLIMMIX). Values for hemoglobin are means ± SDs (number of participants analyzed for the outcome); values for ZPP, CRP, and AGP are geometric means (95% CIs) [number of participants analyzed for the outcome in question]. For each analysis scenario, values in the same row without a common superscript letter are significantly different at α = 0.05. AGP, α-1 acid glycoprotein; CRP, C-reactive protein; IFA, Iron and Folic Acid group: infants were assigned to no supplements, their mothers were assigned to receive 60 mg Fe/d and 400 µg folic acid/d during pregnancy and 200 mg Ca/d as placebo during the first 6 mo postpartum; iLiNS, International Lipid-based Nutrient Supplements; LNS, Lipid-based Nutrient Supplement group: infants were assigned to receive 20 g Lipid-based Nutrient Supplement/d (designed for infants) containing 6 mg Fe/d from 6 to 18 mo of age, their mothers received 20 g Lipid-based Nutrient Supplement/d (designed for women) with the same micronutrients as the MMN group during pregnancy and the first 6 mo postpartum—both Lipid-based Nutrient Supplement products contained 4 additional minerals (calcium, phosphorus, potassium, and magnesium) as well as macronutrients; MMN, Multiple Micronutrient group: infants were assigned to receive no supplements, their mothers were assigned to receive a multiple micronutrient capsule containing 18 vitamins and minerals, including 20 mg Fe, daily during pregnancy and the first 6 mo postpartum; ZPP, zinc protoporphyrin.

2 *P* values compare the means or geometric means of 3 groups, with Tukey–Kramer adjustment for pairwise comparisons.

In the unadjusted analyses of binary outcomes (**Table 3**), there was a trend (*P* = 0.07) towards a lower prevalence of elevated ZPP in the LNS group than in the other 2 groups at 18 mo of age when groups were based on supplements women were intended to receive when enrolled; when groups were based on supplements women actually received when enrolled, the prevalence of elevated ZPP at 18 mo of age was significantly lower in the LNS group than in the other 2 groups, which did not differ in this outcome regardless of how groups were analyzed. The intervention groups did not differ in any of the other binary outcomes. In the secondary analysis (**Supplemental Table 2**), children in the LNS group had a marginally lower prevalence of anemia (38.7% compared with 44.9%; *P* = 0.06) based on the WHO cutoff, and a significantly lower RR (95% CI) of elevated ZPP [0.79 µmol/mol heme (0.64, 0.97 µmol/mol heme); *P* = 0.02] at 18 mo of age compared with the IFA and MMN groups combined.

**TABLE 3:** Unadjusted binary outcome measures (anemia and biomarkers of iron status) for infants in the iLiNS-DYAD randomized trial of daily nutrient supplementation in a semiurban setting in Ghana, by intervention group^1^

	Intervention groups based on supplements mothers were intended to receive when enrolled				Intervention groups based on supplements mothers actually received when enrolled			
	IFA (*n* = 441)	MMN (*n* = 439)	LNS (*n* = 440)	*P* ^2^	IFA (*n* = 441)	MMN (*n* = 439)	LNS (*n* = 440)	*P* ^2^
Anemia^3^								
6 mo	37.1 (31.9, 42.6) [310]	35.4 (30.4, 40.7) [325]	33.5 (28.5, 39.0) [313]	0.65	33.4 (28.4, 38.9) [308]	38.8 (33.7, 44.2) [327]	33.5 (28.5, 39.0) [313]	0.26
18 mo	42.6 (37.4, 48.0) [333]	47.3 (41.9, 52.7) [328]	38.7 (33.6, 44.1) [328]	0.09	43.9 (38.6, 49.4) [321]	45.9 (40.6, 51.2) [340]	38.7 (33.6, 44.1) [328]	0.16
Anemia^4^								
6 mo	19.7 (15.6, 24.5) [310]	19.4 (15.4, 24.1) [325]	16.6 (12.9, 21.2) [313]	0.55	19.2 (15.1, 23.9) [308]	19.9 (15.9, 24.6) [327]	16.6 (12.9, 21.2) [313]	0.54
18 mo	6.0 (3.9, 9.1) [333]	4.9 (3.0, 7.8) [328]	4.6 (2.8, 7.5) [328]	0.68	5.6 (3.6, 8.7) [321]	5.3 (3.4, 8.2) [340]	4.6 (2.8, 7.5) [328]	0.83
Elevated ZPP^5^								
6 mo	31.7 (26.6, 37.2) [300]	37.7 (32.5, 43.1) [316]	35.8 (30.5, 41.4) [299]	0.28	34.6 (29.4, 40.1) [301]	34.9 (29.8, 40.4) [315]	35.8 (30.5, 41.4) [299]	0.95
18 mo	35.3 (30.3, 40.6) [329]	34.7 (29.7, 40.0) [323]	27.5 (22.9, 32.7) [320]	0.07	36.6 (31.5, 42.0)^b^ [317]	33.4 (28.6, 38.7)^ab^ [335]	27.5 (22.9, 32.7)^a^ [320]	0.046
IDA^6^								
6 mo	19.0 (14.9, 23.8) [300]	18.7 (14.7, 23.4) [316]	19.7 (15.6, 24.6) [299]	0.94	17.9 (14.0, 22.7) [301]	19.7 (15.7, 24.5) [315]	19.7 (15.6, 24.6) [299]	0.82
18 mo	23.4 (19.1, 28.3) [329]	21.4 (17.2, 26.2) [323]	18.1 (14.3, 22.7) [320]	0.25	23.3 (19.0, 28.3) [317]	21.5 (17.4, 26.2) [335]	18.1 (14.3, 22.7) [320]	0.26
IDA^7^								
6 mo	12.0 (8.8, 16.2) [300]	11.7 (8.6, 15.8) [316]	12.0 (8.8, 16.2) [299]	0.99	11.6 (8.5, 15.8) [301]	12.1 (8.9, 16.2) [315]	12.0 (8.8, 16.2) [299]	0.98
18 mo	4.9 (3.0, 7.8) [329]	3.4 (1.9, 6.0) [323]	3.4 (1.9, 6.1) [320]	0.55	4.4 (2.6, 7.3) [317]	3.9 (2.3, 6.6) [335]	3.4 (1.9, 6.1) [320]	0.82

1 All supplements were intended for daily consumption. Results are based on logistic regression models (SAS PROC GLIMMIX). Values are percentages (95% CIs) of participants identified as “yes” for the outcome in question [number of participants analyzed for the outcome in question]. Values in the same row without a common superscript letter are significantly different at α = 0.05. IDA, iron deficiency anemia; IFA, Iron and Folic Acid group: infants were assigned to receive no supplements, their mothers were assigned to receive 60 mg Fe/d and 400 µg folic acid/d during pregnancy and 200 mg Ca/d as placebo during the first 6 mo postpartum; iLiNS, International Lipid-based Nutrient Supplements; LNS, Lipid-based Nutrient Supplement group: infants were assigned to receive 20 g Lipid-based Nutrient Supplement/d (designed for infants) containing 6 mg Fe/d from 6 to 18 mo of age, their mothers received 20 g Lipid-based Nutrient Supplement/d (designed for women) with the same micronutrients as the MMN group during pregnancy and the first 6 mo postpartum—both LNS products contained 4 additional minerals (calcium, phosphorus, potassium, and magnesium) as well as macronutrients; MMN, Multiple Micronutrient group: infants were assigned to receive no supplements, their mothers were assigned to receive a multiple micronutrient capsule containing 18 vitamins and minerals, including 20 mg Fe, daily during pregnancy and the first 6 mo postpartum; ZPP, zinc protoporphyrin.

2 *P* values compare all 3 groups, with Tukey–Kramer adjustment for pairwise comparisons.

3 Anemia defined as blood hemoglobin <110 g/L (33).

4 Anemia defined as blood hemoglobin <105 g/L for children at 6 mo of age (34), and blood hemoglobin <100 g/L for children at 18 mo of age (31, 34).

5 Elevated ZPP considered indicative of iron deficiency was defined as ZPP >70 µmol/mol heme. This (moderate) cutoff is consistent with ZPP concentration >10th percentile for preschool children (35–37).

6 IDA was defined as blood hemoglobin <110 g/L and ZPP >70 µmol/mol heme (33, 35–37).

7 IDA was defined as blood hemoglobin <105 g/L (34) and ZPP >70 µmol/mol heme (33, 35–37) for children at 6 mo of age, and blood hemoglobin <100 g/L (31, 34) and ZPP >70 µmol/mol heme (33, 35–37) for children at 18 mo of age.

The results were unchanged in the adjusted analyses (data not shown), except for the prevalence of elevated ZPP, in which case the difference between the LNS group and the other 2 groups became nonsignificant, although the point estimates were consistent with the unadjusted results. Finally, in the sensitivity analysis in which Hb and ZPP values were corrected for inflammation in a subsample (**Supplemental Table 3**), the geometric mean (95% CI) ZPP concentration at 18 mo of age was significantly lower (*P* = 0.044) in the LNS group [53.9 µmol/mol heme (50.7, 57.3 µmol/mol heme)] than in the IFA [59.6 µmol/mol heme (56.2, 63.3 µmol/mol heme)] or the MMN [59.5 µmol/mol heme (56.0, 63.1 µmol/mol heme)] group, whereas the IFA and MMN groups did not differ in this outcome. None of the binary outcome measures generated from the inflammation-corrected Hb and ZPP values (**Supplemental Table 4**) differed between the 2 intervention groups.

## Discussion

We found that supplementation of women's diet with 60 mg Fe/d and 400 µg folic acid/d during pregnancy and placebo in the first 6 mo postpartum, or multiple micronutrient supplements or SQ-LNSs containing 20 mg Fe/d during both periods, did not result in differences in infant Hb or iron status at 6 mo of age. However, infants who consumed SQ-LNSs from 6 to 18 mo of age, after their mothers had been given SQ-LNSs during pregnancy and lactation, had greater iron supply for Hb synthesis and marginally lower prevalence of anemia than their counterparts who did not consume any supplements from 6 to 18 mo of age and whose mothers consumed iron and folic acid during pregnancy only, or multiple micronutrients during pregnancy and lactation.

We previously reported the main weaknesses of our study, including the inability to blind the study women and fieldworkers to who was in the LNS group (in which women and children received SQ-LNSs) and who was in the non-LNS groups (in which women received IFA or MMN capsules and children received no supplementation), assessment of adherence to supplement intakes via maternal reporting rather than through direct observation, and the exposure of 340 women in the IFA and MMN groups to unintended supplements during all or part of pregnancy (12, 13). Further, determining CRP and AGP in a subsample may have limited our ability to detect significant differences among groups in the sensitivity analysis in which we corrected for the effect of inflammation on the Hb and iron status outcomes.

The study, however, had several strengths, such as using a fully randomized design, having active control groups, and complete blinding of the laboratory workers involved in sample collection and analysis. Our approach of measuring anemia by using various Hb cutoffs was an added strength of the study. As previously reported (12), the unintended exposure to IFA or MMN supplements occurred on only 13% of follow-up days, and no women in the LNS group were exposed to any other supplement apart from the intended SQ-LNSs. We compared several secondary outcomes simultaneously, and it is possible that some of our findings may be due to chance because of multiple testing (46). However, these outcomes were prespecified, measured at the same time, and highly correlated, and it was logical that they would be analyzed together (47). Under these circumstances, correcting for multiplicity may be unnecessary and counterproductive (46).

We chose ZPP concentration (i.e., ZPP:H ratio, expressed as µmol/mol heme) as the indicator of children's iron status because it measures the adequacy of iron supply to the bone marrow for Hb synthesis (48), and is able to detect iron deficiency in the bone marrow at early stages (49, 50). In infants, iron stores are often low or depleted (48) because of the high iron requirement (51) for growth and development, and in addition, iron stores may be depleted before absorption is sufficiently upregulated.

It is noteworthy that infants in the 3 groups did not differ in mean Hb concentration or iron status at 6 mo of age. We previously reported (14) that mothers of infants in the MMN and LNS groups, who consumed supplements with a lower iron content (20 mg/d) than did those in the IFA group (60 mg/d), had a lower mean Hb concentration, lower iron status (higher mean ZPP and plasma transferrin receptor), and a higher prevalence of anemia at 36 weeks of gestation. Therefore, the infants in the MMN and LNS groups were expected to be more likely than those in the IFA group to be anemic and/or to have lower iron status (e.g., higher mean ZPP concentration) by 6 mo of age, because of evidence that an infant's risk of developing anemia after birth is related to the mother's anemia and iron status at delivery (52–54), whereas maternal iron intake postpartum does not affect breast-milk iron content (55). On the other hand, infants in the LNS group were previously reported to have a greater mean birth weight than those in the IFA group (13), and thus may have had greater iron stores at birth (52, 56). Our findings suggest that any risk of developing anemia among children in the LNS group (and possibly the MMN group) during the first 6 mo after delivery as a result of their mothers consuming supplements with lower iron content during pregnancy may have been offset through greater iron stores resulting from increased birth weight. Few randomized controlled trials have examined the impact of prenatal iron supplementation on the Hb concentration or iron status of the offspring during the first 6 mo of life (57). Our study provides evidence that in this setting where iron deficiency in infancy is common (58), the consumption of SQ-LNSs during pregnancy (in comparison with the standard dose of iron and folic acid) does not compromise the Hb production or iron status of infants at 6 mo of age. The extent to which the increased birth weight in the LNS group contributed to the Hb concentration and iron status at 6 mo of age requires further investigation.

From 6 to 18 mo of age, the iron dosage (6 mg/d) given to infants in the LNS group was lower than that used in most home fortification trials (59) including our previous study in Ghana (26). However, a similar dosage was used in SQ-LNS products in a trial in Burkina Faso (60), where infants consuming these products from 9 to 18 mo of age along with malaria and diarrhea treatment had lower prevalence of anemia and iron deficiency than did children who received no intervention. In China, the consumption of a fortified food supplement containing 6 mg Fe/d by infants 4–12 mo of age increased the children's mean Hb concentration compared with the consumption of an unfortified food supplement (61). Thus, it is likely that the iron dosage used in the present study was adequate.

Our results on infants’ iron status at 18 mo of age are consistent with those from Burkina Faso (60) and our previous study in Ghana (62) showing that SQ-LNS supplementation reduced the prevalence of iron deficiency when compared with no supplementation for infants. In those 2 previous studies, the percentage of children with low ferritin or elevated serum transferrin receptor was lower among those who consumed SQ-LNSs than among children who did not consume any supplements, although the geometric mean ZPP concentration did not differ between the intervention and nonintervention groups in Burkina Faso (60), and ZPP was not measured in Ghana (62). A previous meta-analysis demonstrated that home fortification of complementary foods, including the use of SQ-LNSs, is effective for the prevention of IDA (59). In the current study, there were no significant differences in mean Hb concentration or prevalence of anemia, which appears to contradict previous results (60, 62). However, there was a trend for a difference in anemia prevalence at 18 mo of age when comparing the LNS group with the other 2 groups combined, equivalent to a 14% reduction in the risk of anemia. A possible reason for the lack of significant group differences could be that in the current trial, children's mean Hb concentration at 6 mo of age (113 g/L) was relatively high, compared with that (107 g/L) in the previous study in Ghana (62) or that (88 g/L) of the 9-mo-old children in Burkina Faso (60). Thus, infants in this study may have been less likely to demonstrate a response to SQ-LNSs than infants in the other 2 studies (60, 62).

According to the Ghana Demographic and Health Survey (GDHS) 2014 report (63), 78% of children 12–17 mo of age and 74% of children 18–23 mo of age in Ghana were anemic when using the WHO cutoff (Hb <110 g/L) to define anemia. The relatively low anemia prevalence at 18 mo of age observed in our trial (42.9%) therefore conflicts with the GDHS 2014 report, perhaps because of regional and/or subregional variations in the distribution of anemia prevalence in Ghana, or methodological differences such as collection of capillary as opposed to venous blood, use of different models of the Hemocue photometer, or different protocols for handling samples for measuring Hb concentration. It is unlikely that the discrepancy is due to a decreasing secular trend of anemia prevalence in Ghana, because the GDHS and our trial were conducted around the same period of time.

Our observation that nearly 39% of the children in the LNS group were anemic at 18 mo of age based on the WHO cutoff of 110 g/L, despite the SQ-LNS supplementation, warrants further discussion. In West Africa including Ghana, the main causes of anemia in children <5 y of age are iron deficiency, malaria, schistosomiasis, sickle cell disorders, and hookworm infection (in decreasing order of importance) (64). We do not have data on sickle cell disorders, but given the low percentage (∼1% at 6 or 18 mo of age) of children who tested positive for malaria, as well as the children's young age of 18 mo, it is unlikely that malaria, schistosomiasis, and hookworm infections contributed substantially to anemia in our sample. Thus, nonresponsive anemia in the LNS group might be due to poor iron absorption or utilization attributable to factors including: antinutritional compounds, e.g., phenolic compounds in the predominantly plant-based diet reducing iron absorption (65–68); gastric acid hyposecretion (69, 70) reducing iron absorption (71, 72); and hepcidin-induced inhibition of iron absorption and transport due to inflammation (73, 74). Alternatively, the WHO cutoff may be too high, leading to an overestimation of anemia prevalence. When using a lower cutoff of <100 g/L, as recommended by INACG (31), anemia prevalence in the LNS group at 18 mo of age was only 4.6%. In Ghana, anemia prevalence in children (using the standard cutoff) has remained high (63) and supposedly nearly unresponsive to various malaria and helminth reduction interventions (75).

We conclude that maternal and infant supplementation with SQ-LNSs increases infants’ iron status in this semiurban setting in Ghana. More research is needed to investigate the lack of Hb response to micronutrient supplementation among children in this and similar settings, and to identify appropriate cutoffs for defining anemia in children (76).

## Supplementary Material

nxy225_Supplemental_File

## Notes

Supported by Bill & Melinda Gates Foundation grant OPP49817 to the University of California, Davis (to KGD).

Author disclosures: SA-A, RTY, AL, HO, PA, UA, BMO, MA, and KGD, no conflicts of interest.

Supplemental Tables 1–4 are available from the “Supplementary data” link in the online posting of the article and from the same link in the online table of contents at https://academic.oup.com/jn/.

Abbreviations used: AGP, α-1 glycoprotein; CRP, C-reactive protein; GDHS, Ghana Demographic and Health Survey; Hb, hemoglobin; IDA, iron deficiency anemia; IFA, iron and folic acid; iLiNS, International Lipid-based Nutrient Supplements; INACG, International Nutritional Anemia Consultative Group; LNS, lipid-based nutrient supplement; MMN, multiple micronutrient; SQ-LNS, small-quantity lipid-based nutrient supplement; ZPP, zinc protoporphyrin.
