# Supplementary material for: Maternal and Infant Supplementation with Small-Quantity Lipid-Based Nutrient Supplements Increases Infants’ Iron Status at 18 Months of Age in a Semiurban Setting in Ghana: A Secondary Outcome Analysis of the iLiNS-DYAD Randomized Controlled Trial
Source: J Nutr. 2019 Jan 8;149(1):149–58. doi: 10.1093/jn/nxy225 (PMC6351141; doi:10.1093/jn/nxy225)
Supplement: nxy225_Supplemental_File [file nxy225_supplemental_file.pdf]

Supplementary data

SUPPLEMENTAL TABLE 1

Unadjusted continuous outcome measures (hemoglobin and biomarkers of iron status and inflammation) for infants in the iLiNS-DYAD randomized trial of daily nutrient supplementation in a semi-urban setting in Ghana, by IFA and MMN groups combined versus LNS group[1]

	IFA+MMN[2] [N=880]	LNS[2] [N=440]	Mean difference or ratio of log means (95% CI)[3]	P[4]
Hemoglobin, g/L				
6 mo	113 ± 10 [635]	114 ± 10 [313]	0.5 (-0.8, 1.9)	0.46
18 mo	112 ± 11 [661]	113 ± 10 [328]	1.2 (-0.1, 2.6)	0.08
ZPP, µmol/mol heme				
6 mo	62.1 (59.7, 64.7) [616]	63.5 (60, 67.1) [299]	1.02 (0.95, 1.10)	0.55
18 mo	59.6 (57.1, 62.1) [652]	53.9 (50.7, 57.3) [320]	0.90 (0.84, 0.97)	0.008
CRP, mg/L[5]				
6 mo	0.45 (0.35, 0.6) [202]	0.41 (0.28, 0.6) [101]	0.90 (0.59, 1.44)	0.66
18 mo	0.64 (0.49, 0.83) [202]	0.94 (0.63, 1.42) [100]	1.47 (0.93, 2.34)	0.10
AGP, g/L[5]				
6 mo	0.85 (0.81, 0.9) [202]	0.8 (0.74, 0.86) [101]	0.93 (0.86, 1.02)	0.15
18 mo	0.95 (0.9, 1) [202]	1.03 (0.96, 1.11) [100]	1.08 (0.99, 1.18)	0.07

[1]IFA+MMN = Iron-Folic Acid and Multiple Micronutrients groups combined: infants received no supplementation from 6 to 18 mo of age, but their mothers were intended to receive 60 mg iron and 400 µg folic acid/d during pregnancy and 200 mg/d calcium as placebo during the first 6 mo postpartum, or a multiple micronutrient capsule containing 18 vitamins and minerals (including 20 mg/d iron) during pregnancy and the first 6 mo postpartum; LNS = Lipid-based nutrient supplement (LNS) group: infants received LNS (designed for infants) containing 6 mg/d iron from 6 to 18 mo of age, their mothers received LNS (designed for women) with the same micronutrients as the MMN group, plus 4 more minerals (Ca, P, K and Mg) as well as macronutrients during pregnancy and the first 6 mo postpartum. All supplements were intended for daily consumption. AGP, alpha-1 acid glycoprotein; CRP, C-reactive protein; ZPP, zinc protoporphyrin. *N* = total number of participants. Results are based ANOVA (SAS PROC GLIMMIX).

[2]Values for hemoglobin are mean ± SD [number of participants analyzed for the outcome]; values for ZPP, CRP and AGP are geometric mean (95% CI) [number of participants analyzed for the outcome in question]

Supplementary data

[3]Values for hemoglobin are mean difference (95% CI); values for ZPP, CRP and AGP are ratio of log-transformed means (95% CI).

[4]P-values compare means or geometric means of two groups.

[5]The sub-sample for the CRP and AGP analyses was selected from the children whose mothers were not pregnant during the period when the temporary mislabeling occurred.

Supplementary data

SUPPLEMENTAL TABLE 2

Unadjusted binary outcome measures (biomarkers of anemia and iron status) for infants in the iLiNS-DYAD randomized trial of daily nutrient supplementation in a semi-urban setting in Ghana, by IFA and MMN groups combined versus LNS group[1]

	IFA+MMN[2] [N=880]	LNS[2] [N=440]	P[3]	Relative Risk (95% CI)[4]
Anemia[5]				
6 mo	36.2 (32.6, 40.0) [635]	33.5 (28.5, 39.0) [313]	0.42	0.93 (0.77, 1.12)
18 mo	44.9 (41.2, 48.8) [661]	38.7 (33.6, 44.1) [328]	0.06	0.86 (0.73, 1.01)
Anemia[6]				
6 mo	19.5 (16.6, 22.8) [635]	16.6 (12.9, 21.2) [313]	0.28	0.85 (0.63, 1.14)
18 mo	5.4 (4.0, 7.5) [661]	4.6 (2.8, 7.5) [328]	0.56	0.84 (0.47, 1.51)
Elevated ZPP[7]				
6 mo	34.7 (31.1, 38.6) [616]	35.8 (30.5, 41.4) [299]	0.76	1.03 (0.85, 1.24)
18 mo	35.0 (31.4, 38.7) [652]	27.5 (22.9, 32.7) [320]	0.020	0.79 (0.64, 0.97)
Iron deficiency anemia[8]				
6 mo	18.8 (15.9, 22.1) [616]	19.7 (15.6, 24.6) [299]	0.75	1.05 (0.79, 1.39)
18 mo	22.4 (19.4, 25.8) [652]	18.1 (14.3, 22.7) [320]	0.13	0.81 (0.62, 1.06)
Iron deficiency anemia[9]				
6 mo	11.9 (9.5, 14.7) [616]	12.0 (8.8, 16.2) [299]	0.93	1.02 (0.70, 1.48)
18 mo	4.1 (2.9, 6.0) [652]	3.4 (1.9, 6.1) [320]	0.60	0.83 (0.42, 1.65)

[1]IFA+MMN = Iron-Folic Acid and Multiple Micronutrients groups combined: infants received no supplementation from 6 to 18 mo of age, but their mothers were intended to receive either 60 mg iron and 400 µg folic acid/d during pregnancy and 200 mg/d calcium as placebo during the first 6 mo postpartum, or a multiple micronutrient capsule containing 18 vitamins and minerals (including 20 mg iron) during pregnancy and the first 6 mo postpartum; LNS=Lipid-based Nutrient Supplement (LNS) group: infants were assigned to receive 20 g/d LNS (designed for infants) containing 6 mg/d iron from 6 to 18 mo of age, their mothers received 20 g/d LNS (designed for women) with the same micronutrients as the MMN group during pregnancy and the first 6 mo postpartum. Both LNS products contained 4 additional minerals (Ca, P, K and Mg) as well as macronutrients. All supplements were intended for daily consumption. ZPP, zinc protoporphyrin. *N*= total number of participants in the group in question Results are based logistic regression models (SAS PROC GLIMMIX).

[2]Data are percentage of participants identified as “yes” for the outcome in question (95% CI) [number of participants analyzed for the outcome in question].

[3]P-values compare all three groups, with Tukey-Kramer adjustment for pairwise comparisons.

[4]Relative risks and 95% CIs were calculated by using Poisson regression (1)

[5]Anemia defined as blood hemoglobin < 110 g/L(2).

Supplementary data

[6]Anemia defined as blood hemoglobin <105 g/L for children at 6 mo of age (3), and blood hemoglobin <100 g/L for children at 18 mo of age (4).

[7]Elevated ZPP considered indicative of iron deficiency was defined as ZPP >70 µmol/mol heme. This (moderate) cutoff point is consistent with ZPP concentration >10th percentile for preschool children (5-7).

[8]Iron deficiency anemia was defined as blood hemoglobin < 110 g/L (2) and ZPP > 70 µmol/mol heme (5-7).

[9]Iron deficiency anemia was defined as blood hemoglobin < 105 g/L (3) and ZPP > 70 µmol/mol heme (5-7) for children at 6 mo of age, and blood hemoglobin < 100 g/L (4) and ZPP > 70 µmol/mol heme (5-7) for children at 18 mo of age.

Supplementary data

SUPPLEMENTAL TABLE 3

Unadjusted hemoglobin and ZPP concentrations corrected for inflammation (CRP and AGP) for a sub-sample of infants in the iLiNS-DYAD randomized trial of daily nutrient supplementation in a semi-urban setting in Ghana, by intervention group[1]

Outcome variable	Intervention groups based on supplements mothers were *intended to receive* when enrolled			
	IFA[2]	MMN[2]	LNS[2]	P[3]
Hemoglobin, g/L				
6 mo	116 ± 10 [102]	115 ± 9 [100]	117 ± 8 [101]	0.49
18 mo	111 ± 11 [101]	111 ± 10 [101]	113 ± 10 [100]	0.31
ZPP, µmol/mol heme				
6 mo	60.8 (57.4, 64.5) [102]	63.4 (56.0, 67.1) [100]	63.5 (60.0, 67.1) [101]	0.47
18 mo	59.6 (56.2, 63.3) [101][b]	59.5 (56.0, 63.1) [101][b]	53.9 (50.7, 57.3) [100][a]	0.044

[1]IFA= Iron+Folic Acid group: infants were assigned to no supplements, their mothers were assigned to receive 60 mg iron and 400 µg folic acid/d during pregnancy and 200 mg/d calcium as placebo during 6 mo postpartum; MMN=Multiple Micronutrient group: infants were assigned to receive no supplement, their mothers were assigned to receive a multiple micronutrient capsule/d containing 18 vitamins and minerals (including 20 mg iron/d during pregnancy and the first 6 mo postpartum; LNS=Lipid-based Nutrient Supplement (LNS) group: infants were assigned to receive 20 g/d LNS (designed for infants) containing 6 mg/d iron from 6 to 18 mo of age, their mothers received 20 g/d LNS (designed for women) with the same micronutrients as the MMN group during pregnancy and the first 6 mo postpartum. Both LNS products contained 4 additional minerals (Ca, P, K and Mg) as well as macronutrients. All supplements were intended for daily consumption. AGP, alpha-1 acid glycoprotein; CRP, C-reactive protein; ZPP, zinc protoporphyrin. The sub-sample included in the CRP and AGP analyses was selected from the children whose mothers were not pregnant during the period when the temporary mislabeling occurred. Correction for inflammation was accomplished by grouping children into 3 inflammation categories (reference (normal CRP and AGP), incubation (raised CRP and normal AGP) and early (raised CRP and AGP) or late (normal CRP and raised AGP) convalescence), estimating the correction factor (CF) for each inflammation category, and multiplying the hemoglobin and ZPP values of each child by the inflammation category-specific CF (8). *N*= total number of participants in the group in question. Results are based on ANOVA (SAS PROC GLIMMIX).

Supplementary data

[2]Values for hemoglobin are Mean ± SD (number of participants analyzed for the outcome); values for ZPP are geometric mean (95% CI) [number of participants analyzed for the outcome]. Values with different superscript letters are significantly different.

[3]P-values compare means or geometric means of 3 groups, with Tukey-Kramer adjustment for pairwise comparisons.

Supplementary data

SUPPLEMENTAL TABLE 4

Unadjusted binary outcome measures (biomarkers of anemia and iron status) corrected for inflammation (CRP and AGP) for a sub-sample of infants in the iLiNS-DYAD randomized trial of daily micronutrient supplementation in a semi-urban setting in Ghana, by intervention group[1]

	Intervention groups based on supplements mothers were *intended to receive* when enrolled			
	IFA[2]	MMN[2]	LNS[2]	P[3]
Anemia[4]				
6 mo	27.5 (19.6, 36.9) [102]	29.0 (20.9, 38.7) [100]	20.8 (13.9, 29.8) [101]	0.37
18 mo	47.5 (38.0, 57.3) [101]	49.5 (39.9, 59.2) [101]	41.0 (31.8, 50.9) [100]	0.45
Anemia[5]				
6 mo	10.8 (6.1, 18.5) [102]	13.0 (7.7, 21.2) [100]	7.9 (4.0, 15.1) [101]	0.51
18 mo	7.9 (4.0, 15.1) [101]	7.9 (4.0, 15.1) [101]	4.0 (1.5, 10.2) [100]	0.45
Elevated ZPP[6]				
6 mo	28.4 (20.5, 38.0) [102]	32.0 (23.6, 41.8) [100]	27.7 (19.8, 37.3) [101]	0.78
18 mo	29.7 (21.6, 39.3) [101]	25.7 (18.1, 35.2) [101]	23.0 (15.8, 32.3) [100]	0.56
Iron Deficiency Anemia[7]				
6 mo	15.7 (9.8, 24.1) [102]	15.0 (9.2, 23.4) [100]	10.9 (6.1, 18.6) [101]	0.57
18 mo	20.8 (13.9, 29.8) [101]	16.8 (10.7, 25.5) [101]	18.0 (11.6, 26.8) [100]	0.76
Iron Deficiency Anemia[8]				
6 mo	6.9 (3.3, 13.7) [102]	9.0 (4.7, 16.4) [100]	5.9 (2.7, 12.6) [101]	0.70
18 mo	5.0 (2.1, 11.4) [101]	3.0 (1.0, 8.8) [101]	4.0 (1.5, 10.2) [100]	0.77

[1]IFA= Iron+Folic Acid group: infants were assigned to receive no supplements, their mothers were assigned to receive 60 mg iron and 400 µg folic acid/d during pregnancy, and 200 mg/d calcium as placeboduring 6 mo postpartum; MMN=Multiple Micronutrient group: infants were assigned to receive no supplements, their mothers were assigned to receive a multiple micronutrient capsule/d containing 18 vitamins and minerals (including 20 mg iron/d during pregnancy and the first 6 mo postpartum; LNS=Lipid-based Nutrient Supplement (LNS) group: infants were assigned to receive 20 g/d LNS (designed for infants) containing 6 mg/d iron from 6 to 18 mo of age, their mothers received 20 g/d LNS (designed for women) with the same micronutrients as the MMN group during pregnancy and the first 6 mo postpartum. Both LNS products contained 4 additional minerals (Ca, P, K and Mg) as

well as macronutrients. All supplements were intended for daily consumption. AGP, alpha-1 acid glycoprotein; CRP, C-reactive protein; ZPP, zinc protoporphyrin. The sub-sample included in the CRP and AGP analyses was selected from the children whose mothers were not pregnant during the period when the temporary mislabeling occurred. Correction for inflammation was accomplished by grouping children into 3 inflammation categories (reference (normal CRP and AGP), incubation (raised CRP and normal AGP) and early (raised CRP and AGP) or late (normal CRP and raised AGP) convalescence), estimating the correction factor (CF) for each inflammation category, and multiplying the hemoglobin and ZPP values of each child by the inflammation category-specific CF (8). N= total number of participants in the group in question. Results are based logistic regression models (SAS PROC GLIMMIX).

[2]Data are percentage of participants identified as “yes” for the outcome in question (95% CI) [number of participants analyzed for the outcome]. For each analysis scenario, values in the same row and have different superscript letters are significantly different at alpha = 0.05.

[3]P-values compare all three groups, with Tukey-Kramer adjustment for pairwise comparisons.

[4]Anemia defined as blood hemoglobin < 110 g/L (2).

[5]Anemia defined as blood hemoglobin <105 g/L for children at 6 mo of age (3), and blood hemoglobin <100 g/L for children at 18 mo of age (4).

[6]Elevated ZPP considered indicative of iron deficiency was defined as ZPP >70 µmol/mol heme. This (moderate) cutoff point is consistent with ZPP concentration >10th percentile for preschool children (5-7).

[7]Iron deficiency anemia was defined as blood hemoglobin < 110 g/L (2) and ZPP > 70 µmol/mol heme (5-7).

[8]Iron deficiency anemia was defined as blood hemoglobin < 105 g/L (3) and ZPP > 70 µmol/mol heme (5-7) for children at 6 mo of age, and blood hemoglobin < 100 g/L (4) and ZPP > 70 µmol/mol heme (5-7) for children at 18 mo of age.

Supplementary data

References

1. Spiegelman D, Hertzmark E. Easy SAS calculations for risk or prevalence ratios and differences. American journal of epidemiology. 2005;162:199-200.
2. WHO. Haemoglobin concentrations for the diagnosis of anaemia and assessment of severity (WHO/NMH/NHD/MNM/11.1) [Internet]. Vitamin and Mineral Nutrition Information System. Geneva, World Health Organization [cited 2018 May 19]. Available from: http://www.who.int/vmnis/indicators/haemoglobin.pdf. 2011.
3. Domellof M, Dewey KG, Lonnerdal B, Cohen RJ, Hernell O. The diagnostic criteria for iron deficiency in infants should be reevaluated. J Nutr. 2002;132:3680-6.
4. Nestel P, INACG Steering Committee. Adjusting Hemoglobin Values in Program Surveys [Internet]. INACG, Washington, DC [cited 2018 May 19]. Available from: http://pdf.usaid.gov/pdf_docs/PNACQ927.pdf. 2002.
5. Crowell R, Ferris AM, Wood RJ, Joyce P, Slivka H. Comparative effectiveness of zinc protoporphyrin and hemoglobin concentrations in identifying iron deficiency in a group of low-income, preschool-aged children: practical implications of recent illness. Pediatrics. 2006;118:224-32.
6. Rettmer RL, Carlson TH, Origenes ML, Jack RM, Labb RF. Zinc protoporphyrin/heme ratio for diagnosis of preanemic iron deficiency. Pediatrics. 1999;104:e37.
7. Soldin OP, Pezzullo JC, Hanak B, Miller M, Soldin SJ. Changing trends in the epidemiology of pediatric lead exposure: interrelationship of blood lead and ZPP concentrations and a comparison to the US population. Ther Drug Monit. 2003;25:415-20.
